# Epidemiology of animal bite in Iran during a 20-year period (1993–2013): a meta-analysis

**DOI:** 10.1186/s41182-019-0182-5

**Published:** 2019-11-29

**Authors:** Maliheh Abedi, Amin Doosti-Irani, Fatemeh Jahanbakhsh, Amirhossein Sahebkar

**Affiliations:** 1 Pasteur Institute of Iran, Center for Reference and Research on Rabies, Tehran, Iran; 2 Department of Epidemiology, School of Public Health, Hamadan University of Medical Sciences, Hamadan, Iran; 3 Halal Research Center of IRI, FDA, Tehran, Iran; 4 Biotechnology Research Center, Pharmaceutical Technology Institute, Mashhad University of Medical Sciences, Mashhad, Iran; 5 Neurogenic Inflammation Research Center, Mashhad University of Medical Sciences, Mashhad, Iran

**Keywords:** Rabies, Epidemiology, Animal bite, Incidence, Iran

## Abstract

**Background:**

Rabies is a fatal disease that still kills 2–6 people a year in Iran. A meta-analysis was conducted in order to generate accurate data on animal bite exposure, and to estimate the incidence of animal bite across the country.

**Materials and methods:**

Major national and international electronic databases were searched using the keywords “animal bite,” rabies, prevalence, incidence, and Iran. Web of Knowledge, PubMed, Scopus, Ovid, and ScienceDirect were used as international databases, and the national databases included Science Information Database, MagIran, and IranDoc. Descriptive cross-sectional studies addressing the incidence of animal bite were selected and screened by two authors, and pre-specified data were extracted. The population of provinces or cities of studies was extracted from the Statistical Centre of Iran. The overall incidence of animal bite in Iran was estimated using a random-effects model with 95% confidence interval (CI). Study quality was assessed using the STROBE recommended checklist.

**Results:**

A total of 34 studies were selected for the meta-analysis out of 1215 retrieved studies. The number of animal bites in the studies during 1993–2013 was 230,019 cases. The overall estimated incidence rate of animal bite in Iran was 13.20/1000 (95%, CI 12.10, 14.30) and the mean age of people was 26.23 (SD = 5.02) year. The incidence rate of animal bite among males (14.90/1000) was much higher than females (4.55/1000), and was higher in rural areas (17.45/1000) compared with urban areas (4.35/1000). The incident rate was highest among students compared with other reported occupations. The incidence rate of dogs was 10.40/1000 followed by cats, cows, wolves, jackals, and foxes. Domestic animals had a higher incidence rate than stray and wild animals. The incidence rate of animal bite during spring was 4.90/1000; however, the incidence rate in other seasons had no significant difference. In the retrieved studies, the highest incidence rate of animal bite was found in the West Azerbaijan Province (146.83/1000).

**Conclusion:**

The current study is the first comprehensive analysis of the published animal bite studies in Iran. Accurate data on animal bite incidence may lead to more effective policy-decisions towards more efficient resource allocation to primary health care for reducing rabies case. Such information is a primary and major necessity for rabies control program in the country. Animal bite reduction can significantly minimize the risk of rabies infection, thereby reducing public health costs for the expensive post-exposure treatment.

## Introduction

Rabies is a fatal anthropozoonotic viral disease that kills around 60,000 people worldwide annually, with over 99% of all human rabies deaths occurring in the developing world [1–3]. With the exception of the Antarctic, rabies is widely distributed across the world [4]. People of all age groups are susceptible but children are at greater risk [5]. Rabies has two epidemiological cycles, an urban and a sylvatic cycle. Dogs are the main reservoir in the urban cycle, the predominant cycle in the Middle East. In some regions including South East Asia and the Middle East, sylvatic and urban cycle operate simultaneously, making rabies epidemiology more complex [4].

Globally around 3.7 million DALYs (disability-adjusted life years) are estimated to be lost due to rabies annually [6]. Human rabies is one of the most severe infectious diseases, and one of the most neglected tropical diseases due its direct mortality and DALYs lost [7, 8]. Children are a vulnerable population and are at a higher risk of exposure to rabid animals, mostly dogs; typically 30–50% of those exposed to rabid dogs are children under 16 years old [5]. Rabies affects poor rural communities; about 84% of human rabies deaths occur in rural areas [8].

Asia bears the highest burden of rabies. Around 60% of all human rabies deaths and DALYs lost are seen in Asia [6]. According to the World Health Organization (WHO) estimates, 31,000 human rabies deaths occur annually in Asia which comes to about 56% of total global rabies deaths [8]. The main route of transmission is through rabid dog bites, which account for 96% of human rabies cases [4]. In Iran the main route of transmission is through domestic dog bite [9].

Pre- or post-exposure (PrEP and PEP) vaccination is the only treatment of this crucial disease; however it inflicts a great economic cost to the health system of the countries [7]. Annually the overall economic costs of human rabies estimate as 8.6 billion USD (95% CIs 2.9–21.5 billion) with the largest proportion of costs due to premature death in Asia and Africa [6].

Rabies is a serious enzootic disease and public health problem in Middle East especially in Iran, Jordan, Syria, and Lebanon [10]. Beside the absence of accurate data in this region, the scale of the rabies burden in Middle East has not been investigated previously and little information is available on rabies in this region. Based on available literature and population data it is estimated that 350 deaths occur (95% CI, 270–450) and 13,100 DALYs are lost (95% CI, 11,100–15,900) in the Middle East per annum [8]. Annually 12 million people received PEP in Asia that cost USD $563 million [3]. In Iran, the Ministry of Health and Education spends 11,301,336,400 Rials (1,052,756.1 USD; USD was equal 10,735 Rials at 21 September 2011; Source: Iran’s Central Bank) annually to provide PEP program. In addition the overall cost of treating each case of animal bite and each prevented death was estimated to be 1,589,275 Rials (148.04 USD) and 63,824,117 Rials (5945.42 USD) [11].

Rabies is one of the most important zoonotic diseases endemic to wildlife populations in Iran, and frequently occurs in domestic livestock as well. It is prevalent in almost all 31 provinces of country [12–14]. Though the human rabies mortality rate decreased from 0.9 per million people in the 1980s to 0.02–0.03 per million people in recent years, it still kills 2–6 persons every year and the disease has not been brought under control. The number of people receiving PEP has more than doubled between 1997 and 2009, which shows the need for urgent action in the country [12].

Accurate data on animal bite incidence may lead to more effective policy-decisions towards more efficient resource allocation to primary health care for reducing human and animal rabies cases in the country. Lack of accurate data on rabies burden and animal bite exposure are the major factors that cause low commitment to rabies control in Iran. Several sporadic studies have been performed to estimate the incidence rate of animal bite and epidemiology of rabies in some provinces or cities, but the results have been contradictory and not sufficient for estimating rabies incidence in the entire country. The Center for Disease Control of Iran Ministry of Health collects data and statistics on animal bite annually, but as this data has not been published, we decided to analyze articles, which reported animal bite locally during 1993–2013.

In this study, a meta-analysis was conducted to estimate the incidence of animal bite across the country.

## Material and methods

### Definition

Based on World Health Organization protocols on rabies control and management, animal bite is divided in three different categories: (i) is contact with animal through feeding; no treatment is required for such bites if reliable history of the animal is provided. (ii) is peck the bare skin, small scratches, abrasion without bleeding and licking of wounds. In these cases, immediate prophylaxis treatment is needed, though treatment may be discontinued if the animal is under supervision for 10 days and appears healthy or if animal was euthanized and the specific test of rabies was negative. (iii) is one or more animal bite or deep cutaneous scratches or saliva infection of mucous membrane (licking). In this group, the urgent prescription of prophylaxis and immunoglobulin treatment is required. Similar to the second group, in this group the treatment could be discontinued. These three categories that reported as animal bite in the studies were used for rabies PEP across the country [15].

### Searching

The following major electronic databases: Web of Knowledge, PubMed, Scopus, Ovid and Science Direct, as well as the national databases Science Information Database, MagIran, and IranDoc, were searched with the following keywords: animal bite, rabies, prevalence, incidence, and Iran. The reference lists of all available studies were checked to obtain additional literature. In addition, data from the first and second International World Rabies Day seminars and Iranian veterinary congress were searched.

### Inclusion criteria

All descriptive studies regarding the incidence of animal bite including rabies in Iran during 1993–2013 were considered irrespective of publication language and date. We decided to choose this period because there is poor understanding of the disease burden in Iran during these years. Several sporadic studies were performed in this period in high-incidence regions, but the results have been contradictory and not sufficient to estimate rabies incidence in the country. Therefore, there is a need to pool the data to obtain an overall estimate. Furthermore, the Disease Control Center of Iran Ministry of Health has data related to rabies and yearly animal bite, but this information is not published. We therefore decided analyze this time period as it seems to be lacking information. In recent years (from 2014 up to now), these data are becoming more available because of international seminars and meetings. Hence, this choice may fill a gap in national rabies control programs. The studies reporting animal bite in cases other than rabies were excluded.

### Extraction of population data

For estimating the total incidence rate, the population of each province or city was required. The population was specifically mentioned in 10 studies, and for the other 24 studies, population data was obtained from the publication information base Statistical Centre of Iran [16]. Although we needed the population of males and females in rural and urban areas of each province or city of studies to obtain the incidence rate in location and gender subgroups. We extracted all the population of studies in these subgroups from the publication information base Statistical Centre of Iran [16].

### Data collection and quality assessment

Two authors screened all the retrieved studies by their title and abstract independently, and after that they reviewed full texts to extract the accurate studies, which included the required criteria for meta-analysis. Then, any disagreement of the authors was discussed and after rechecking, 100% agreement was reached. The variables that were extracted for data analysis included location and year of study, population, total number of animal bite, gender, age, rural, urban, bite site (including leg and hand), occupation (including student, farmer, and housewife), animal type (including dog, cat, wolf, fox, jackal, and cow), animal dependency (including domestic, wild, and stray), dog dependency (including domestic and stray), season (including spring, summer, autumn, and winter), type of the injury (including superficial and deep), and complete prophylaxis treatment.

For quality assessment of the reporting, seven items were selected from the STROBE recommended checklist [17]. The studies were assessed for quality based on whether they (1) present the demographic characteristic of participants, i.e., age, sex, residency, and occupation; (2) report number of outcome animal bite; (3) explain a precise definition of animal bite; (4) describe how the sampling was performed; (5) mention the type of study; and (6, 7) give accurate date and location of study. The studies were classified as following order: the studies that fulfilled entire criteria were assorted as high quality. The studies that did not contain one criterion were classified as intermediate quality. The studies that did not have more than one criterion were classified as low quality.

As some important items were required for analysis, the studies that have no clear data on number of animal bite, location, and date were excluded from the analysis.

### Heterogeneity and statistical analysis

We conducted the incidence rate of animal bite in this study by dividing the number of animal bite cases into the mean population of the year(s) from census results in each study. The statistical heterogeneity was evaluated using the chi-square test at 10% significant level. In addition, the heterogeneity across the results of the included studies was quantified by I2 statistic, and the between study variance was estimated using tau-square (Tau2) statistic [18, 19]. The statistical software Stata 11 (Stata Corp, College Station, TX, USA) was used for data analysis. Meta-analysis was conducted to obtain the overall incidence of animal bite in Iran using a random-effects model [20] with 95% confidence interval (CI). Sub-group analysis was performed based on quality of included studies, geographic region of Iran, gender, location of living (rural or urban regions), and year of study conduction.

## Result

### Retrieving studies

We retrieved 1215 studies, including 1107 studies from international electronic databases and 108 studies through national electronic databases and no new studies from checking reference lists and contacting with authors. The general characteristic of studies including author, year of publication, location, number of animal bite, population, and incidence rate are shown in Table 1.

**Table 1:** General characteristics of retrieved studies

Author	Year of publish	Location	Animal bite cases	Population	Incidence rate
Alavi, S. M.	2008	Khuzestan Province	44,088	4,480,000●	9.84
Alavinia, SM.	2015	North Khorasan Province	18,571	839,650	22.12
Amiri, M.	2009	Shahroud	588	241,742	2.43
Babaeeian-Moghaddam, M.	2015	Ghouchan	814	179,714	4.53
Bahonar, A.R.	2008	Ilam Province	2431	510,157	4.76
Behnampour,N.	2011	Aq-Qala	13,142	110,953	118.45
Bijari, B.	2011	Birjand	1662	270,000●	6.16
Dadypour, M.	2009	Kalaleh	3496	152,568●	22.91
Dehghani, R.	2013	Semirom	1246	75,636●	16.47
Eslamifar, A.	2008	Tehran	8806	7,569,282	1.16
Feizhaddad,M.A.	2014	Shush	1300	190,728	6.82
Ghafouri, M.	2015	Bojnourd	5909	349,270	16.92
Sabouri Ghannad, M.	2014	Borujerd	2783	320,547●	8.68
Havasian, M.R.	2015	Ilam	598	213,579	2.80
Kassiri, H.	2012	Ahvaz	2390	1,272,378	1.88
Kassiri, H.	2014	Shush	2282	193,469	11.80
Kassiri, H.	2014	Ahvaz	4186	1,295,200	3.23
Kassiri, H.	2014	Islam-Abad Gharb	2864	171,300	16.72
Khazaei, S.	2014	Tuyserkan	425	103,786	4.09
Majidpour, A.	2004	Ardebil Province	4331	1,191,618	3.64
Mohtasham-Amiri, Z.	2015	Gilan Province	1771	2,480,874●	0.71
Naghibi, S.A.	2014	Mazandaran Province	25,869	2,935,032	8.81
Najafi, N.	2009	Mazandaran Province	23,168	3,000,000●	7.72
Rafiei, N.	2014	Aq-Qala District	1025	119,423	8.58
Ranjbar, H. M.	2006	Torbat Heydariyeh	1038	360,341	2.88
Rezaeinasab, M.	2007	Kerman Province	21,546	2,166,572	9.95
Riyahi,S.M	2012	Tabas	480	68,949	6.96
Sabouri Ghannad, M.	2012	Ilam Province	4420	555,517●	7.96
Sadeghi, A.	2003	West Azarbayjan Province	3867	26,335.9●	146.83
Saghafipour, A.	2014	Qom Province	7246	1,109,336	6.53
Sheikholeslami, N. Z.	2009	Rafsanjan	1542	250,000●	6.17
Taghvaii, M. R. E.	2013	Mashhad	14,037	2,904,910	4.83
Vahdati, S.	2013	Tabriz	1084	1,656,447	0.65
Zohrevandi, B.	2014	Rasht	1014	918,445	1.10

*95% confidence interval

● Population was reported in the desired study

**Fig. 1:**
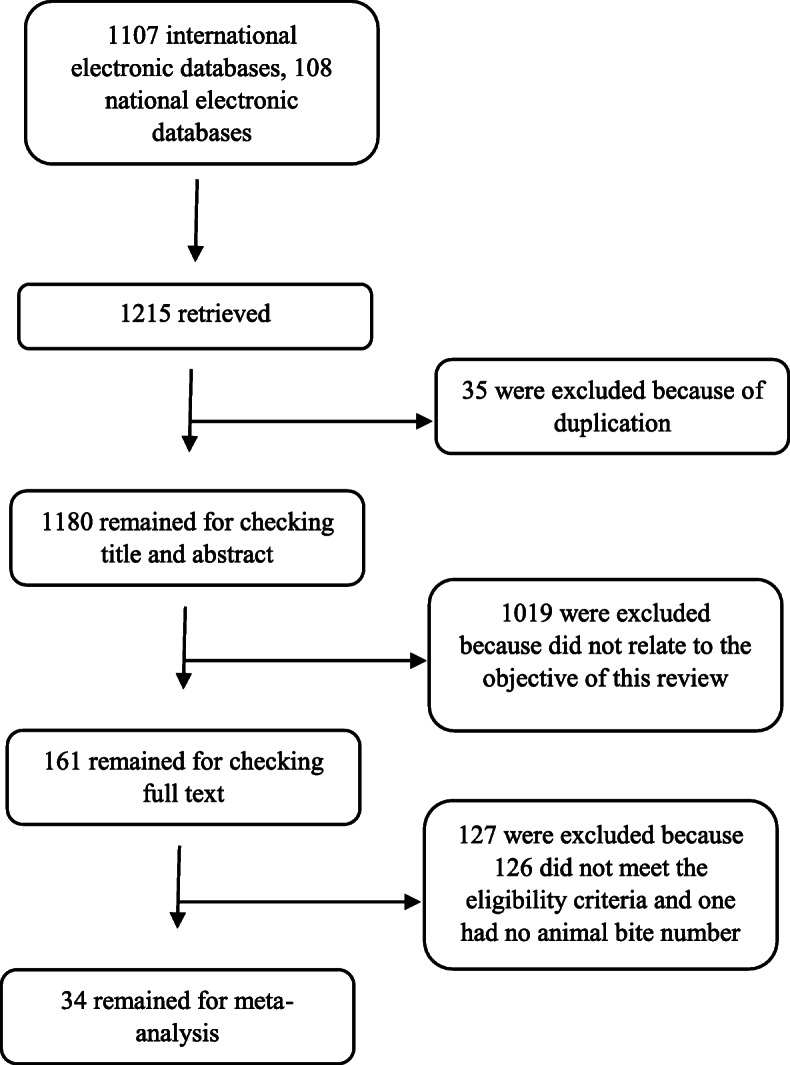
Total number of retrieved studies, deletion procedure of non-related studies and number of remain articles for meta-analysis

Of all 1215 references, 35 references were excluded as duplicates, 1019 references did not relate to the objective of this review, 126 references did not meet the eligibility criteria after reading full text of studies, and one reference had no animal bite number. Eventually we included 34 studies in the meta-analysis. The procedure of selection to obtain the retrieved studies for meta-analysis is shown in Fig. 1.

### Quality assessment

The studies were divided into three categories based on the quality of reporting using STROBE checklist of items as follows: 16 studies (47.05%) had all criteria and classified as high quality; 16 studies (47.05%) intermediate quality; and 2 studies (5.9%) had low quality.

### Incidence rate

The total number of animal bite reported in the 34 studies was 230,019 cases. All incidence rates were estimated in 1000 population. The overall incidence rate of animal bite in Iran was 13.20 per 1000 population (95% CI 12.10, 14.30) and the mean age based on the 25 studies that reported age was 26.23 (SD = 5.02) year. In the case of gender, the incidence rate in males was 14.90 per 1000 (95% CI 13.70, 16.10) and in females was 4.55 per year (95% CI 4.20, 5.00). One study did not report the number of animal bite in male and gender subgroups. In 33 studies which reported the number of animal bite in location subgroup, the rural area had a higher incidence rate than urban area. The incidence rate in rural area was 17.45 per year (95% CI 15.60, 19.30), and in urban area was 4.35 (95% CI 3.90, 4.75).

The incidence rate of animal bite in leg was 7.00 per year (95% CI 6.50, 7.40) which was higher than hand 2.30 per year (95% CI 2.00, 2.60) in bite site subgroup. The incidence rate of animal bite among student 2.80 per year (95% CI 2.45, 3.00) was the highest in the reported occupation, followed by housewife 2.10 per year (95% CI 1.80, 2.40) and farmer 1.80 per year (95% CI 1.55, 2.00). Dog in particular owned dog had the highest incidence rate among the other animals that caused animal bite. The incidence rate of dog was 10.40 per year (95% CI 9.70, 11.00) and domestic dog was 7.15 per year (95% CI 4.80, 9.00). The incidence rate of animal bite in the springtime was 4.90 per year (95% CI 4.30, 5.45) that is noticeably different from other seasons. The incidence rate of animal bite caused by domestic animal was 6.90 per year (95% CI 4.80, 9.00) and superficial wounds was 4.40 per year (95% CI 3.55, 5.20). Eventually the incidence rate of incomplete prophylaxis treatment was more than 8 per 1000 higher than complete type. The incidence rate of incomplete vaccination was 11.80 per year (95% CI 9.40, 14.15) and complete vaccination was 3.65 per year (95% CI 3.15, 4.15).

All the incidence rate of variables, confidence interval (95% CI), *P* value, and the number of studies which reported the variable are shown in Table 2.

**Table 2:** The incidence rate of variables in related subgroup

Group	Incidence (per 1000)	95% CI	*P* value*	Number of studies
Bite	13.20	12.10, 14.30	< 0.001	34
Gender				
Male	14.90	13.70, 16.10	< 0.001	33
Female	4.55	4.20, 5.00	< 0.001	33
Location				
Rural	17.45	15.60, 19.30	< 0.001	33
Urban	4.35	3.90, 4.75	< 0.001	33
Bite Side				
Leg	7.00	6.50, 7.40	< 0.001	28
Hand	2.30	2.00, 2.60	< 0.001	27
Occupation				
Student	2.80	2.45, 3.00	< 0.001	21
Housewife	2.10	1.80, 2.40	< 0.001	15
Farmer	1.80	1.55, 2.00	< 0.001	15
Animal type				
Dog	10.40	9.70, 11.00	< 0.001	33
Cat	0.65	0.50, 0.70	< 0.001	28
wolf	0.035	0.025, 0.05	< 0.001	13
Fox	0.015	0.001, 0.02	< 0.001	12
Jackal	0.023	0.015, 0.03	< 0.001	11
Cow	0.35	0.25, 0.50	0.001	5
Animal dependency				
Domestic	6.90	4.80, 9.00	< 0.001	13
Wild	0.30	0.10, 0.45	< 0.001	12
Stray	1.00	0.70, 1.30	< 0.001	9
Dog dependency				
Domestic dog	7.15	5.20, 9.10	< 0.001	6
Stray dog	0.55	0.35, 0.75	< 0.001	6
Season				
Spring	4.90	4.30, 5.45	< 0.001	19
Summer	3.35	2.85, 3.80	< 0.001	17
Fall	3.47	3.04, 3.90	< 0.001	18
Winter	3.40	2.90, 3.90	< 0.001	17
Bite type				
Deep	0.65	0.55, 0.80	< 0.001	15
Superficial	4.40	3.55, 5.20	< 0.001	15
Prophylaxis treatment				
Complete	3.65	3.15, 4.15	< 0.001	16
Incomplete	11.80	9.40, 14.15	< 0.001	15

*P value for test of heterogeneity

### Incidence rate by area

We divided studies in area 1 (north and south) and area 2 (west and east) for further analysis. The numbers of studies which belong to north were 23 and south were 11, although 19 studies were divided in to west and 15 to east.

Since the heterogeneity was too high (100%), we could not pool the data of studies and meta-analysis not applicable. In the retrieved studies the highest incidence rate of animal bite belonged to West Azarbayjan Province (146.83/1000) in North-West. The highest incidence rate in North-East was 118.45/1000 and 22.91 in Aq-Qala and Kalaleh. In addition, the highest incidence rate of animal bite in south of country was seen 11.80/1000 in Shush county. The incidence rates of all retrieved studies in descending order are shown in Table 3.

**Table 3:** The incidence rates of studies in descending order

	Study	Location	Area	Incidence rate	95% CI
1	Sadeghi, A. 2003	West Azarbayjan	North-West	146.83	142.20, 151.46
2	Behnampour, N. 2011	Aq-Qala	North-East	118.45	116.42, 120.47
3	Dadypour, M. 2009	Kalaleh	North-East	22.91	22.16, 23.67
4	Alavinia, SM. 2015	North Khorasan	North-East	22.12	21.799, 22.436
5	Ghafouri, M. 2015	Bojnourd	North-East	16.92	16.487, 17.350
6	Kassiri, H. 2014	Islam-Abad Gharb	West	16.72	16.11, 17.33
7	Dehghani, R. 2013	Semirom	Center	16.47	15.559, 17.388
8	Kassiri, H. 2014	Shush	South-West	11.80	11.31, 12.28
9	Rezaeinasab, M. 2007	Kerman Province	South	9.95	9.81, 10.08
10	Alavi, S. M. 2008	Khuzestan	South-West	9.84	9.75, 9.93
11	Naghibi, S.A. 2014	Mazandaran	North	8.81	8.71, 8.92
12	Sabouri Ghannad, M. 2014	Borujerd	West	8.68	8.36, 9.00
13	Rafiei, N. 2014	Aq-Qala	North	8.58	8.06, 9.11
14	Sabouri Ghannad, M. 2012	Ilam Province	West	7.96	7.72, 8.19
15	Najafi, N. 2009	Mazandaran	North	7.72	7.62, 7.82
16	Riyahi, S.M. 2012	Tabas	East	6.96	6.34, 7.58
17	Feizhaddad, M.A. 2014	Shush	South-West	6.82	6.45,7.19
18	Saghafipour, A. 2014	Qom Province	North-West	6.53	6.38, 6.68
19	Sheikholeslami, N. Z. 2009	Rafsanjan	South	6.17	5.86, 6.48
20	Bijari, B. 2011	Birjand	East	6.16	5.86, 6.45
21	Taghvaii, M. R. E. 2013	Mashhad	North-East	4.83	4.75, 4.91
22	Bahonar, A.R. 2008	Ilam Province	West	4.76	4.58, 4.96
23	Babaeeian-Moghaddam, M. 2015	Ghouchan	North-East	4.53	4.22, 4.84
24	Khazaei, S. 2014	Tuyserkan	North-West	4.09	3.71, 4.48
25	Majidpour, A. 2004	Ardebil Province	North-West	3.64	3.53, 3.74
26	Kassiri, H. 2014	Ahvaz	South-West	3.23	3.13, 3.33
27	Ranjbar, H. M. 2006	Torbat Heydariyeh	North-East	2.88	2.70, 3.06
28	Havasian, M.R. 2015	Ilam	West	2.80	2.58, 3.02
29	Amiri, M. 2009	Shahroud	North-East	2.43	2.24, 2.63
30	Kassiri, H. 2012	Ahvaz	South-West	1.88	1.80, 1.95
31	Eslamifar, A. 2008	Tehran	North-West	1.16	1.14, 1.19
32	Zohrevandi, B. 2014	Rasht	North-West	1.10	1.04, 1.17
33	Mohtasham-Amiri, Z. 2015	Gilan	North-West	0.71	0.68, 0.75
34	Vahdati, S. 2013	Tabriz	North-West	0.65	0.62, 0.69

## Discussion

The total number of animal bite reported in the 34 studies during the 20-year period from 1993 to 2013 was 230,019 cases. In the present study the overall estimated incidence rate of animal bite in Iran was 13.20/1000 (95%, CI 12.10, 14.30) and the mean age of total people who had animal bite injuries across the country was 26.23 years. Our study showed that the incidence rate of animal bite in males (14.90/1000) was much higher than females (4.55/1000) and in rural areas (17.45/1000) was 13/1000 more than urban areas (4.35/1000). The incidence rate of bites in lower extremities (leg, 7.00/1000) is more prominent than upper extremities (hand, 2.30/1000) in our study. The incident rate of bites in student was 2.80/1000 as the highest reported occupation, followed by housewife (2.10/1000) and farmer (1.80/1000). Owned dogs had highest incidence rate of animal bite, and generally bites by dogs were the most common (10.40/1000) followed by cat (0.65/1000), cow (0.35/1000), wolf (0.03/1000), jackal (0.02/1000), and fox (0.01/1000). In addition, the incidence rate of domestic and stray dog was 7.15/1000 and 0.55/1000 respectively. The incidence rate of spring in our study was 4.90/1000; however, in other seasons it had no significant difference with autumn 3.47/1000, winter 3.40/1000, and summer 3.35/1000.The present study indicated that the incidence rate of domestic animal (6.90/1000) was higher than stray (1.00/1000) and wild (0.30/1000) animals. As the rate of domestic animal was more frequent, the incidence rates of superficial wound (4.40/1000) and incomplete prophylaxis (11.80/1000) were more than deep wound (0.65/1000) and complete vaccination (3.65/1000). In the retrieved studies, the highest incidence rate of animal bite was seen in West Azerbaijan Province (146.83/1000) which is located in North West of country.

The overall estimated incidence rate of animal bite was 13.20/1000 (95%, CI 12.10, 14.30). Considering high number of the rabies control centers (700) and huge amount of the health budget in the country that is used for PEP, human rabies cases are quite low in comparison with the high incidence rate of animal bite. Compared with Pakistan, the country’s neighbor to the east, that has 7–9.8 human cases per million [21], Iran has 0.02 per million which is quite similar to Turkey [12]. The incidence rate of human rabies in Karachi in Pakistan ranged from 7.0 to 9.8 cases per million per year which was consistent with the high incidence of rabies in South-East Asia [21]. WHO expert consultation on rabies reported that the incidence of human rabies deaths in some endemic regions was estimated to be 1.1–1.8/100,000 in rural Bangladesh, 2.5–7.5/100,000 in Bhutan, and 2.8–11.5/100,000 in Cambodia [8]. These data are high in comparison with Iran as an endemic region of rabies.

Rabies is a disease of young people mostly children younger than 15 years old in Asia and Africa as a frequent vulnerable population (30–50%) [5, 22]. The current study estimated that the mean age of total people who had animal bite injuries across the country was 26.23 (SD 5.02) which represents a significant percentage of young people and children in different studies conducted in the country, as seen in Kerman Province which indicated the most people (25.76%) who had animal bite was 10–19 years old [23]. Similarly, the study in Oman showed that commonly affected age group was the 10–19-year-old people. Children have a more playful nature and tend to play more with animals, besides less defense power, which cause them as a victim of animal bites [24].

The incidence rate of animal bite in males (14.90/1000) was much higher than females (4.55/1000) which demonstrated similarity to all studies conducted in different parts of country. It is not surprising that the higher ratio (3:1) of the human rabies also was reported among men in the country [25]. The higher animal bite incidence rate in males may be due to frequent outdoor activity and close contact to animals particularly in rural areas and risk behavior for encountering with them. On the other hand, this proportion has shown similarity to study in Oman as another country of Middle East and contradictory from animal bite reports in United States of America (USA) and Puerto Rico which are located in other parts of global. These reports indicated that most of cases in Oman were males [24]; females had more bite injuries than males in USA, because of the higher rate of keeping pets at home among females [26].

WHO second report on rabies declared more than 84% human rabies of Asia and Africa occur in rural and poor areas [8]. The incidence rate of animal bite in rural areas (17.45/1000) was 13/1000 more than urban areas (4.35/1000) and indicated that animal bite in rural areas of Iran is more frequent, as 68.75% of human rabies cases were reported from rural areas in the country [25]. Apart from studies, which were performed in cities of country with limited livestock life surrounding, all others showed the higher incidence of animal bite in the rural areas. The study in North Khorasan Province indicated 80.7% of animal bite cases occurred in rural areas [27]; in contrary, the study in Tehran the capital city of Iran showed that the 94% of cases were in urban areas, which is the most populated city in the country [28]. Furthermore, the results of Knobel study predicted that rabies death in rural areas was five times more than urban areas in Asia and Africa [5]. Like the current study, the previous studies in India and Ethiopia confirmed this prediction. In India 75% of cases were in poor and low-income group and 80.3% of this group belong to rural areas and the incidence rate of bite victims in these areas was 18 [29]. In Ethiopia 73.2% of human rabies exposure were reported from rural areas [30].

The incidence rate of lower extremities (leg, 7.00/1000) is prominently higher than upper extremities (hand, 2.30/1000). Most of the human rabies cases in Iran had wounds on heads or faces that is the one of the most risk factors for getting rabies infection via bite [25]. In contrast the upper extremities (48.9%) were more common than lower extremities (35.4%) in Oman [24]. Feet are the nearest part of the body to animals’ mouth (mostly dogs and wild carnivores) when encountered and perhaps because of this reason it had more incidence rate in current study and most of national studies. Hands could be injured due to feeding or stroking animals, and this may cause more common upper extremities in Oman, since cats were most responsible animal (48.3%) in bite injuries in Oman and they have more connection to hands. A study in Gilan Province of Iran showed that most of dog bite cases are injured in lower extremities because many of them were bitten when they were entering to dogs guarding territory and besides cases that had injuries in their upper extremity were significantly older than cases that had injuries in their lower extremity or head, face, and neck [31].

The overall mean age (26.23) of the study indicated that a large percentage of bite victims were children and youth which include the vast majority of students. The incident rate among student was 2.80/1000 as the highest reported occupation, followed by housewife (2.10/1000) and farmer (1.80/1000). In the retrieved studies the reported occupation was different; however, these three types were most indicated. A study in Aq-Qala City showed that 28.7% students, 18% housewives, and 16.8% farmers were the highest occupations [32]. Students spend much time outdoors and the possibility of contact with animals is increased; housewives in livestock areas and cities are often responsible for taking care of animals and pets. A study in Gilan declared that women in rural area of Iran have more connection with their neighbors than men and they go to their neighbors’ houses frequently, so it is common that dogs show territorial aggression when unfamiliar people are entering the house they reside in [31].

Dog bites are the route of rabies transmission to human in Iran. Dog populations are larger in rural area than in urban area, and most of them are guarding dogs and unleashed [12, 31]. The incidence rate of domestic dogs was 7.15/1000 and stray dogs was 0.55/1000. Similarly, in all other epidemiological studies performed in Iran, the percentages of dog’s bite were higher than other animals. In our retrieved studies the most and least percentages of dog’s bite were 97.8% in Aq-Qala district and 61.7% in Ghom Province [32, 33]. In similar study which was conducted in India, 91.5% of bite victim cases occurred with dogs but in contrast most of them (63%) were stray [29]. After dogs, cat’s bites were more frequent (4.7%) and other animals are not common in Iran. In addition, Puerto Rico study reported that 81.1% of animal bite occurred by dogs [34]. In contrast, in Oman the most common animal was cat (48.3%), followed by dog (35.2%) [24]. However, dogs are the prime suspect of causing animal bite globally but the results of studies in different parts may show variance because of different lifestyle and cultural behavior. In Iran, dogs are kept as a guard and sheepdog in villages and as a pet or guard in cities. Therefore, bite injury may occur due to feeding, playing, or even attacking of rabid dog. This explanation could indicate the frequent incidence rate of owned dogs in Iran. Oman research declared that because of religious reason and cultural beliefs dogs are usually not kept as pets and their presence around humans is usually discouraged [24, 31]. Beside dogs in the country wildlife reservoirs have become increasingly important in rabies transmission. Foxes, jackals, wolves, and mongoose have been reported as wildlife reservoirs of rabies around the world [4]. In Iran fox, jackal, and wolves are main wild life animals that transmit the disease; based on unpublished data of Pasteur Institute of Iran, Reference and Research Center on Rabies, gray Indian mongoose and small Indian mongoose are playing a great role in rabies transmission in south of Iran.

The incidence rate of spring in our study was noticeably different from other seasons (4.90/1000). An adverse study in Ethiopia showed that a significant number of rabies exposure cases were reported during fall and winter (30.18%) that is obviously because of geographical and climate difference [30]. In Iran spring with moderate weather is the proper time for people to travel or spend their time outdoors where the presence of animals is more possible. Also a study in Ilam Province reported that the incidence of animal bite in winter could be due to a greater incitement of animals for finding food [35].

The incidence rate of domestic animal (6.90/1000) was higher than stray (1.00/1000) and wild (0.30/1000) animals. As the incidence rate of incomplete vaccination was more frequent (11.80/1000), the incidence rates of domestic animal and superficial wound (4.40/1000) were more. The prophylaxis treatment regimen in Iran is free of charge and 5 doses IM vaccination at days 0, 3, 7, 14, and 28 (Essen), based on WHO protocols on rabies control and management [12, 15]. According to this definition since the incidence rate of domestic animal was more frequent and they were more likely to be healthy, the prophylaxis treatment discontinued and the incidence rate of incomplete vaccination became higher.

**Fig. 2:**
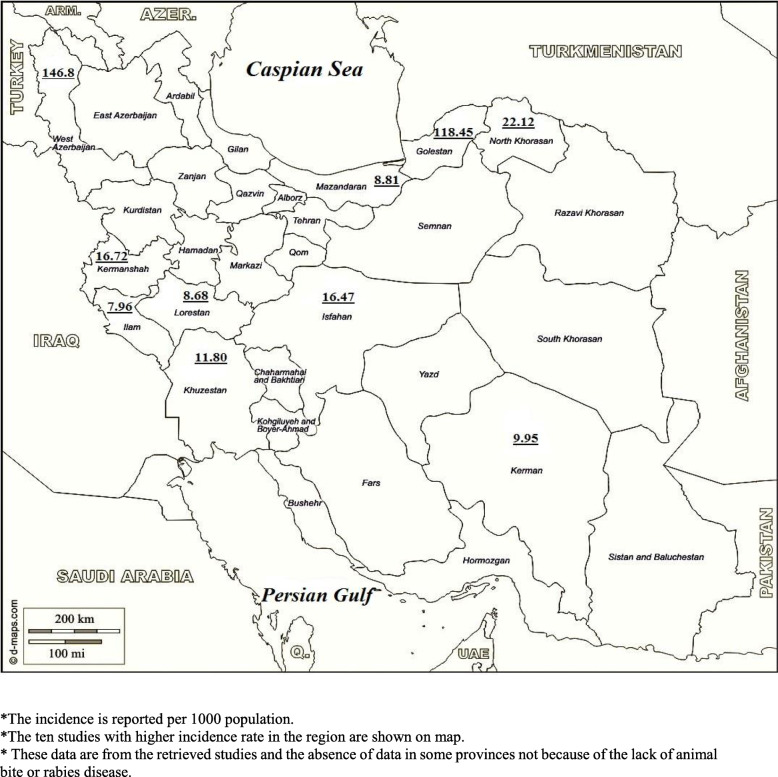
The incidence rate of top ten studies. *The incidence is reported per 1000 population. *The ten studies with higher incidence rate in the region are shown on map. *These data are from the retrieved studies and the absence of data in some provinces not because of the lack of animal bite or rabies disease

We were planning to divide studies in major geographical direction for further analysis; however, because of 100% heterogeneity it could not be possible. Based on the first Meeting of the Middle East and Eastern Europe Rabies Expert Bureau (MEEreb) [12], the most affected provinces are located in the north-east, east, and south of the country. The incidence rates of first ten largest studies in the same area are shown in Fig. 2. As it is seen in this figure, the areas which are located in North-West, North-East had extremely high incidence rates which were much more than other parts of country. These results had similarity and a few differences with MEEREB report. The differences may be due to the lack of sufficient data in all parts of country.

The incidence rate of animal bite among students was the highest among reported occupations. Limited knowledge about dog behavior, inability to protect themselves, not realizing the danger, and not telling their parents of a minor exposure make children at higher risk of disease. For these reasons, increasing educational awareness in children about the risk of rabies exposure resulting from dog bites is critical, and they should be a primary target of rabies prevention efforts. Improving rabies educational awareness among children as well as the public could help to reduce the risk of rabid animal bite in the country. For reaching this goal in the country, comprehensive and appropriate programs in schools and among families should be performed to inform them about all the points associated with the disease like the risk of confronting the disease and its appropriate and timely action after exposure [36]. Besides, since children are the most affected age group of animal bite, educational awareness in school can dramatically reduce the cost of PEP.

Rabies surveillance, collection, and processing of data at the national levels should be done with accuracy. Epidemiological surveillance involves estimation of incidence and prevalence of disease in different regions. The information provides the foundation for planning rabies control measures and to demonstrate the effectiveness of such programs. In the twenty-first century, rabies is beginning to be viewed as a winnable battle as more and more countries understand what is required to prevent human rabies and are outfitting themselves with the knowledge and tools necessary to begin planning their own national rabies control programs [4, 36]. Also the cost of a post-bite treatment in humans is about 20–100 times more than the vaccination of a dog [4]. Therefore, the investment of rabies control programs in dog and wild life reservoir should be designed to reduce the cost of PEP in the country. Required multiplex strategies for rabies control in animals and humans are including interagency cooperation between responsible organizations, communication management, strengthening of rabies surveillance system, identification and data recording, recording and keeping accurate information of animal bites, increasing public awareness, mass vaccination of dogs and wild life reservoirs, stray dog population control, strengthening of laboratory facilities, prompting management of animal bite wounds, and promoting timely and adequate prophylaxis treatment and immunization.

Animal bites and rabies are both under-reported in many developing countries, and there is poor understanding of the disease burden in Iran. Lack of accurate data on this field may lead to high incidence rate of animal bites, which was our main reason for performing this study. While the obtained results are informative, some limitations deserve to be mentioned. Firstly, the retrieved studies might have not been conducted in some data and studies were not from all provinces. However, since the data of studies were extracted from bite management center of the respective areas, the statistical information derived from this analysis can be reliably used for the control and reduction of animal bite and reducing public health costs for treatment of animal bite victims. Another limitation was the descriptive studies and heterogeneity of results, which made meta-analysis of data in some specific areas of the country impossible.

## Conclusion

The current study is the first comprehensive analysis of the published animal bite studies in Iran. Accurate data on animal bites may lead to preventive measures aimed at reducing animal bites and the risk of rabies infection. Such information is a primary and major necessity of rabies control program in the country. As rabies is the most important zoonotic disease in Iran that imposes a considerable cost on the public health system for the PEP, special attention should be paid to the management and control of the disease in the country. Animal bite reduction can significantly minimize the risk of rabies infection and associated healthcare costs for the expensive post-exposure treatment.

## Data Availability

Not applicable.

## Abbreviations

CI: Confidence interval
DALYs: Disability-adjusted life years
MEEreb: Middle East and Eastern Europe Rabies Expert Bureau
PEP: Post-exposure
PrEP: Pre-exposure
WHO: World Health Organization
