# Correction to: Epidemiology of animal bite in Iran during a 20-year period (1993–2013): a meta-analysis

**DOI:** 10.1186/s41182-020-00294-9

**Published:** 2021-01-26

**Authors:** Maliheh Abedi, Amin Doosti-Irani, Fatemeh Jahanbakhsh, Amirhossein Sahebkar

**Affiliations:** 1 Pasteur Institute of Iran, Center for Reference and Research on Rabies, Tehran, Iran; 2 Department of Epidemiology, School of Public Health, Hamadan University of Medical Sciences, Hamadan, Iran; 3 Johns Hopkins University Bloomberg School of Public Health, Baltimore, USA; 4 Halal Research Center of IRI, FDA, Tehran, Iran; 5 Biotechnology Research Center, Pharmaceutical Technology Institute, Mashhad University of Medical Sciences, Mashhad, Iran; 6 Neurogenic Inflammation Research Center, Mashhad University of Medical Sciences, Mashhad, Iran

**Correction to: Trop Med Health 47, 55 (2019)**

**https://doi.org/10.1186/s41182-019-0182-5**

The authors would like to clarify that, at the time the study was conducted and completed, the primary affiliation of Fatemeh Jahanbakhsh was Pasteur Institute of Iran, Center for Reference and Research on Rabies, Tehran, Iran. Fatemeh Jahanbakhsh left the institute in 2018 while the authors were submitting this article [1] for publication and her current affiliation is Johns Hopkins University Bloomberg School of Public Health. The authors would also like to acknowledge that the map presented in the figure of 2 of the article was taken (with modification) from d-maps [2].

## References

[1] Abedi M, Doosti-Irani A, Jahanbakhsh F, Sahebkar A (2019). Epidemiology of animal bite in Iran during a 20-year period (1993–2013): a meta-analysis. Trop Med Health.

[2] https://d-maps.com/carte.php?num_car=329&lang=en.

